# Quality indicators in type 2 diabetes patient care: analysis per care-complexity level

**DOI:** 10.1186/s13098-019-0428-8

**Published:** 2019-05-02

**Authors:** Josiane Schneiders, Gabriela H. Telo, Leonardo Grabinski Bottino, Bruna Pasinato, Jeruza Lavanholi Neyeloff, Beatriz D. Schaan

**Affiliations:** 1 Programa de Pós-Graduação em Endocrinologia, Universidade Federal do Rio Grande do Sul, Rua Ramiro Barcelos 2350, Prédio 21, 6º andar, Porto Alegre, RS 90035-003 Brazil; 2 Hospital de Clínicas de Porto Alegre, Porto Alegre, Brazil

**Keywords:** Type 2 diabetes mellitus, Quality indicators, Primary health care, Tertiary health care

## Abstract

**Background:**

This study was developed to evaluate quality indicators in type 2 diabetes patient care at the Unified Public Health System’s primary and tertiary health care centers within a local population.

**Methods:**

This was a retrospective cohort of 488 patients with type 2 diabetes (148 in each primary health care unit, ESF and UBS, and 192 at the tertiary health care unit) with a 1-year follow-up to evaluate the following care quality indicators: nephropathy, neuropathy and retinopathy tests, yearly lipid profile and nutritional assessments, and an inquiry about tobacco use. The presence of > 50% of the quality of care assessment measures was considered acceptable. Indicators were also evaluated in relation to patients without proper diabetes control (HbA1c > 8.5%).

**Results:**

In the results, a high percentage of patients were excluded specifically for not presenting the two HbA1c tests within a year (n = 208, 58.1% at ESF; n = 225, 58.4% at UBS; and n = 39, 16.9% at the tertiary health care unit). From the included patients, only 7 (4.7%) at ESF, 7 (4.7%) at UBS, and 52 (27.0%) at the tertiary health care unit showed > 50% of the quality criteria covered. When only patients without proper diabetes control were evaluated, none of them at any of the health care units showed all the quality criteria covered.

**Conclusions:**

Our results show a low percentage of care assessment measures at each evaluated health care unit, pointing out the need to improve the protocols and care lines of diabetic patients.

## Background

Type 2 diabetes is a chronic and progressive disease with a high prevalence in the global population [1]. Treatment of this population within a health care system should stratify care based on disease severity and resource requirements; patients deemed “low complexity” should remain in primary care while patients who present metabolic imbalances after several treatment regimens, patients who use complex insulin schemes, or patients with advanced chronic complications who demand highly complex resources for their treatment are referred to institutions capable of meeting their demands [2].

In Brazil, primary care is split into two categories: Family Health Teams (ESF, *Equipe de Saúde da Família*) and Basic Health Care Units (UBS, *Unidade Básica de Saúde*). The ESFs are responsible for coverage of all patients in a predetermined area, and the UBSs also have spontaneous demands and/or demands referred by other services. In this case, there is no ascription of patients, and the delimitation of the coverage area refers exclusively to health surveillance actions. The ESFs are composed of at least one doctor, one nurse, one nursing assistant, and four to six community health agents. The community agents must reside in their respective activity areas to guarantee a bond and similar cultural identity with the families under their responsibility [3]. The ESFs’ promotion of health education in lower socioeconomic environments, where hyperglycemia has a higher prevalence and more consequences, may be a mechanism to address obesity and the diabetes epidemic at the individual level [4]. The UBS teams are formed by doctors (clinicians, pediatricians, and gynecologists-obstetricians), nurses, dentists, nursing assistants, and technical support staff. Other medical specialists make up the staff according to need, such as ophthalmologists, dermatologists, and cardiologists [5]. Tertiary care, also called high complexity care, is responsible for high complexity situations or ones with high technology demands and is suitable for a limited number of health needs [6]. This care is dispensed mostly through hospitals and large centers.

Literature clearly suggests that using diabetes patient care quality indicators improves disease management and reduces related complications and treatment costs [7–12]. The Organization of Cooperation and Economic Development has determined nine diabetes care quality indicators to compare health systems in its member countries. Those indicators are split into three areas: (1) care process [annual glycated hemoglobin (HbA1c) and low density lipoprotein cholesterol (LDL-c) tests as well as annual screenings for nephropathy and retinopathy]; (2) proximal outcome (control of HbA1c and LDL-c); and (3) distal outcome (rate of lower-limb amputations, nephropathy, and cardiovascular mortality) [7]. However, there is a wide variety of proposed indicators, with no agreement on which are most recommended. Some authors also suggest adapting to the sociocultural peculiarities of each population [12].

In Brazilian literature, few studies cover the quality of type 2 diabetes patient care [13–17]. The purpose of this study is to describe and compare, through a set of indicators that are common to the diverse recommendations sources, the quality of type 2 diabetes patient care for people treated at different formats of primary care units (ESF and UBS) and at tertiary care units in Porto Alegre/RS.

## Methods

### Design

This is a cohort study with a 1-year follow-up. Data were collected retrospectively through a medical records analysis.

### Patient identification

The evaluation encompasses patients from primary and tertiary care centers with a follow-up of 1 year or more from October of 2011 to January of 2016. Patients were selected from a primary care health center composed of one ESF and one UBS unit via a manual active search of patient registrations and medical records. For tertiary care, patients were selected via a search of the hospital’s electronic medical records system. At both centers, included patients were aged 18 years or older, had a previous type 2 diabetes diagnosis (with a clearly described history of antidiabetics use), had a follow-up of at least 1 year in their place of attendance (observation period of this cohort), and showed two HbA1c measurements.

The two-measure HbA1c criterion was based on recommendations from the American Diabetes Association (ADA), which considers the minimum criteria necessary to evaluate patients with diabetes [18], and on the patient’s follow-up time in this cohort. When there were several 1-year follow-ups after the first HbA1c in the two care centers, we used the most recent period for this analysis. Patients who were pregnant or who participated in clinical trials during the study follow-up were excluded.

### Data collection

After patient selection, previously trained investigators gathered data through an online form especially designed for this project. Investigators trained the research staff in data collection until an adequate agreement between them was reached (a kappa coefficient > 95% in a pilot with 50 questionnaires among the two researchers). The primary outcome was a composite of quality indicators for type 2 diabetes care that were measured in both groups of patients (primary and tertiary care).

In the study, the following quality indicators were used: nephropathy testing (an appointment with a nephrologist, a creatinine test, or a spot or 24-h sample of urinary albumin); retinopathy evaluation (an appointment with an ophthalmologist or fundoscopy/retinography test results); neuropathy evaluation (a feet assessment, a 10 g monofilament test, or a 128 Hz diapason test); dyslipidemia evaluation [LDL-c measured directly or calculated through total cholesterol, triglycerides and high density lipoprotein cholesterol (HDL-c) test results]; inquiry about tobacco use (active tobacco use registered on patient’s medical records or an inquiry about tobacco use); suspicion of poor adherence to treatment (patient’s medical records register that the patient was not properly following medical treatment recommendations); and nutritional assessment (an appointment/follow-up with a nutritionist registered on the patient’s medical records). When considering quality of care, a team of experts (which included this manuscript’s researchers) considered 50% or more of the care assessment measures evaluated at their health care unit as a minimum acceptable level of patient care quality.

### Statistical analysis

Statistical analysis was performed in SPSS software, version 21. Descriptive data were presented on average and standard deviation. The Chi square test was used for categorical variables. When significant, we used the post hoc proportion comparison test. Analysis of variance (ANOVA) was used to evaluate the statistical significance between the proportions of the three groups (UBS, ESF and tertiary health care unit). When significant, Tukey’s post hoc test was used. A P value less than or equal to 0.05 was considered statistically significant.

### Ethical commitment

This project was approved by the institution’s Ethical and Research Committee under the number 2016-0286 in accordance with the Standard Guidelines and Regulatory Research Involving Human Beings. It was also approved by the National Health Council in accordance with resolution 466/12 and by the Porto Alegre Municipal Health Office. This document follows the STROBE Statement’s checklist of items that should be included in reports of cohort studies [19].

## Results

**Fig. 1:**
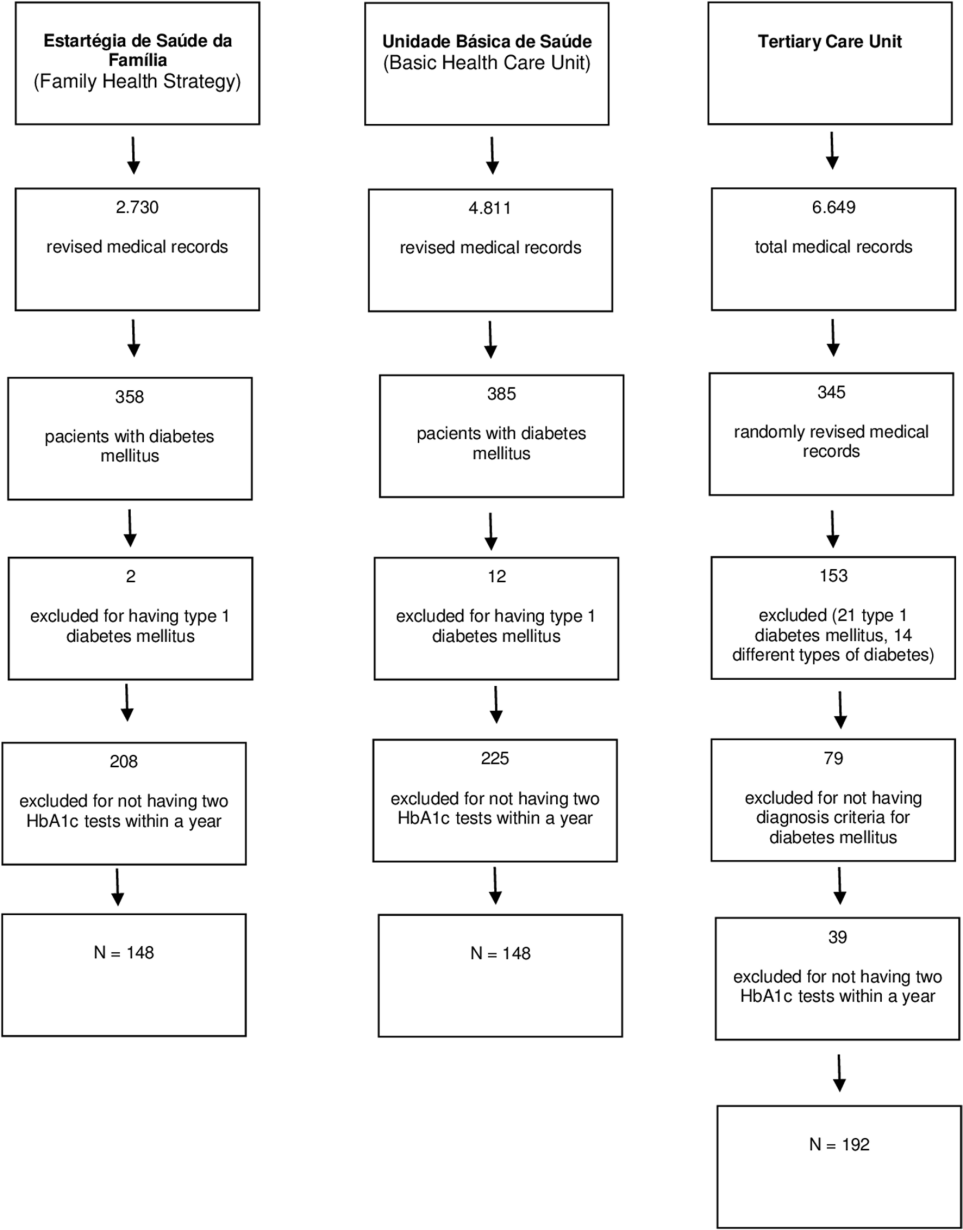
Data collection flowchart

This study included a final sample of 148 patients in each of the two primary health care segments (ESF and UBS) and 192 patients in the tertiary health care unit. The sample selection steps are explained in detail in Fig. 1. From the 14,190 medical records reviewed, 472 were excluded because patients did not have two HbA1c evaluations in the last year. When comparing the included participants with the excluded patients based on the HbA1c criterion, no statistical differences were found with regard to age [70.6 ± 11.2 years at ESF, 69.5 ± 12.8 years at UBS, and 63.8 ± 13.3 years in the tertiary unit (P = 0.32)] or sex (female 57.4%, P = 0.41). Other data were not available for excluded patients (data not shown).

The population’s mean age was 68.5 ± 10.4 at ESF, 68.0 ± 10.6 at UBS, and 63.5 ± 10.2 at the tertiary health care unit, and they were mostly female (59.4%) and Caucasian (89.9%) (Table 1). A minority of this population had graduated from secondary school (21.5%) and was professionally active (21.7%). Regarding comorbidities, a higher percentage of the patients with a follow-up at the tertiary health care unit presented arterial hypertension than patients from the two primary care units (85.8% at ESF, 79.1% at UBS, and 92.7% the tertiary health care unit), but there was no difference regarding dyslipidemia and depression. The complications (macrovascular and microvascular) related to diabetes were most frequently observed in patients at the tertiary health care unit (16.9% at ESF, 12.8% at UBS, and 57.3% at the tertiary health care unit).

**Table 1:** Clinical and demographic data of the baseline population

	ESF	UBS	Tertiary health care	P
	(N = 148)	(N = 148)	(N = 192)	
Sex (% female)	95 (64.2^a^)	93 (62.8^b^)	92 (47.9^b^)	0.003
Skin color (% white)	133 (89.9)	139 (93.9)	167 (87.0)	0.11
Age (years)^1^	68.5^a^ ± 10.4	68.0^a^ ± 10.6	63.5^b^ ± 10.2	< 0.001
Education (% complete secondary school)^2^	53 (37.6^a^)	13 (46.4^a^)	39 (22.2^b^)	0.002
Occupation (% active)	30 (22.4^a^)	11 (45.8^b^)	65 (38.7^b^)	0.004
HbA1c %^2^	7.60^a^ ± 1.7	7.9^a^ ± 1.8	8.6^b^ ± 2.0	< 0.001
Hypertension, n (%)^2^	127 (85.8^ab^)	117 (79.1^b^)	178 (92.7^a^)	0.001
Dyslipidemia, n (%)	102 (68.9)	96 (64.9)	140 (72.9)	0.28
Depression, n (%)	30 (20.3)	26 (17.6)	36 (18.8)	0.84
Diabetes complications, n (%)^2^	25 (16.9^a^)	19 (12.8^a^)	110 (57.3^b^)	< 0.001
Statin, n (%)	102 (68.9)	87 (58.8)	134 (69.8)	0.08
Metformin, n (%)	127 (85.8^a^)	128 (86.5^a^)	144 (75.0^b^)	0.009
NPH insulin, n (%)	31 (20.9^a^)	25 (16.9^a^)	128 (66.7^b^)	< 0.001
Regular insulin, n (%)	2 (1.4^a^)	3 (2.0^a^)	50 (26.0^b^)	< 0.001

Data are shown as n (%) or mean and standard deviation

*ESF* Estratégia de Saúde da Família (Family Health Strategy), *UBS* Unidade Básica de Saúde (Basic Health Care Unit)

^1—a,b,c^Represent statistically different means

^2—a,b,c^Represent statistically different proportions

**Fig. 2:**
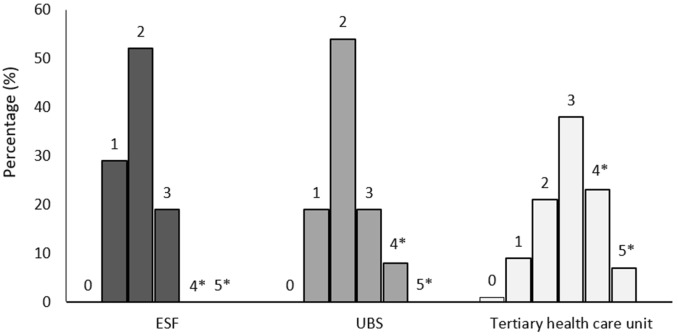
Number (%) of quality indicators covered per type of service. *ESF* Estratégia de Saúde da Família (Family Health Strategy), *UBS* Unidade Básica de Saúde (Basic Health Care Unit). * > 50% of the quality indicators

While evaluating quality indicators in type 2 diabetes patient care, one observation used as an inclusion criterion in this study was the presence of two HbA1c measurements at a 1-year follow-up. At each of the different care levels, a high percentage of patients was excluded specifically for not conforming to this criterion (n = 208, 58.1% at ESF; n = 225, 58.4% at UBS; and n = 39, 16.9% at the tertiary health care unit) (Fig. 1). Among patients included in the final analysis, we observed substantial differences between tertiary and primary care in relation to assessment items for nephropathy (83.1% at ESF, 86.5% at UBS, and 95.8% at the tertiary health care unit), retinopathy (11.5% at ESF, 14.9% at UBS, and 35.9% at the tertiary health care unit), neuropathy (8.8% at ESF, 10.1% at UBS, and 58.9% at the tertiary health care unit) and nutritional assessment (10.1% at ESF, 24.3% at UBS, and 38.0% at the tertiary health care unit), all of which were most frequently assessed at the tertiary health care unit (Table 2). However, with the exception of the assessment items for nephropathy and neuropathy, only a minority of patients showed quality of care assessments, even at the tertiary health care unit. With regard to the inquiry about tobacco use, no difference was found among the health care units, with all of them showing a low covered assessment of this quality of care criterion (8.0%). When we evaluated the total of care assessment measures per health care unit, only 7 patients (4.7%) at ESF, 7 patients (4.7%) at UBS, and 52 patients (27.0%) at the tertiary health care unit presented half or more of the quality criteria covered (Fig. 2).

**Table 2:** Quality indicators in type 2 diabetes patient care per type of service

	ESF	UBS	Tertiary health care	P
	(N = 148)	(N = 148)	(N = 192)	
Nephropathy tests, n (%)	123 (83.1)	128 (86.5)	184 (95.8)	< 0.001
Retinopathy tests, n (%)	17 (11.5^a^)	22 (14.9^a^)	69 (35.9^b^)	< 0.001
Neuropathy tests, n (%)	13 (8.8^a^)	15 (10.1^a^)	113 (58.9^b^)	< 0.001
Nutritional assessment, n (%)	15 (10.1^a^)	36 (24.3^c^)	73 (38.0^b^)	< 0.001
Dyslipidemia tests, n (%)	112 (75.7^a^)	90 (60.8^c^)	106 (55.2^b^)	< 0.001
Inquiry about tobacco use, n (%)	15 (10.1)	11 (7.4)	13 (6.8)	0.50

Data were shown as n (%)

*ESF* Estratégia de Saúde da Família (Family Health Strategy), *UBS* Unidade Básica de Saúde (Basic Health Care Unit)

^a,b,c^Represent statistically different proportions

The mean HbA1c was substantially higher at the tertiary health care unit when compared to the primary care units (7.6 ± 1.7% at ESF, 7.9 ± 1.8% at UBS, and 8.6 ± 2.0% at the tertiary health care unit; P < 0.001). Similarly, the median and the interquartile interval of the amount of HbA1c tests performed during the observation period of this cohort were higher at the tertiary health care unit [2 (2–4) at ESF, 2 (2–4) at UBS, and 3 (2–6) at the tertiary health care unit]. In spite of the higher complexity profile of patients with a follow-up at the tertiary health care unit (according to Table 1), there is a tendency, with no statistical significance, toward poor adherence in primary care units when compared to tertiary health care units (41.2% at ESF, 39.2% at UBS, and 50.0% at the tertiary health care unit; P = 0.10).

**Fig. 3:**
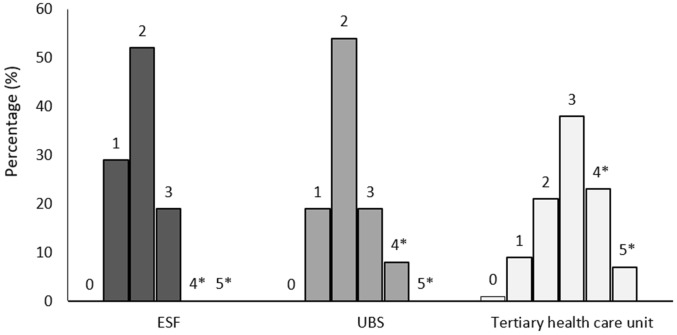
Number (%) of quality indicators covered per type of service for patients with poor glycemic control (HbA1c > 8.5%). *ESF* Estratégia de Saúde da Família (Family Health Strategy), *UBS* Unidade Básica de Saúde (Basic Health Care Unit). * > 50% of the quality indicators

When only patients without proper diabetes control (HbA1c > 8.5%) were considered (n = 154), we found similar results to the total sample. We observed substantial differences between tertiary and primary care in relation to assessment items for nephropathy (higher at the tertiary health care unit), retinopathy (very low at the ESF and higher at the tertiary health care unit), neuropathy (very low at the ESF and higher at the tertiary health care unit), and nutritional assessment (lowest at the ESF and highest at the tertiary health care unit) (Table 3). In both groups, the total sample and the group of patients without proper diabetes control (HbA1c > 8.5%), only a minority of patients had 50% or more of the diabetes care indicators evaluated by their providers. In regards to the inquiry about tobacco use, no difference was found among the health care units, and all of them presented a low covered assessment of this quality of care criterion. When we considered the total assessments indicating the quality of care measures per health care unit, no patient at any health care unit presented all assessments. Also, zero patients at ESF, 3 patients (8.1%) at UBS, and 26 patients (30.2%) at the tertiary health care unit presented half or more of the quality criteria covered (Fig. 3). Among these patients, there was a difference in poor adherence to treatment among the health care units. Poor adherence to treatment was lower at UBS (83.9% at ESF, 70.3% at UBS, and 88.4% at the tertiary health care unit; P = 0.048).

**Table 3:** Quality indicators in type 2 diabetes patient care per type of service with poor glycemic control (HbA1c > 8.5%)

	ESF	UBS	Tertiary health care	P
	(N = 31)	(N = 37)	(N = 86)	
Nephropathy tests, n (%)	25 (80.6^a^)	34 (91.9^ab^)	83 (96.5^b^)	0.02
Retinopathy tests, n (%)	3 (9.7^a^)	5 (13.5^ab^)	29 (33.7^b^)	0.006
Neuropathy tests, n (%)	3 (9.7^a^)	5 (13.5^a^)	54 (62.8^b^)	< 0.001
Nutritional assessment, n (%)	4 (12.9^a^)	11 (29.7^ab^)	32 (37.2^b^)	0.04
Dyslipidemia tests, n (%)	23 (74.2)	22 (59.5)	46 (53.5)	0.13
Inquiry about tobacco use, n (%)	1 (3.2)	3 (8.1)	9 (10.5)	0.53
All the quality indicators, n (%)	0 (0)	0 (0)	1 (0.5)	> 0.999
> 50% of the quality indicators, n (%)	0 (0^a^)	3 (8.1^ab^)	26 (30.2^b^)	< 0.001

Data were shown as n (%)

*ESF* Estratégia de Saúde da Família (Family Health Strategy), *UBS* Unidade Básica de Saúde (Basic Health Care Unit)

^a,b,c^Represent statistically different proportions

## Discussion

This study’s objective was to evaluate care quality indicators in type 2 diabetes patients treated in the Brazilian Public Health System and to compare results at the primary and tertiary health care levels. Our results showed few assessments indicating the quality of care at each of the evaluated health care units. Among the patients included, over 50% of the tertiary health care unit patients and around 15% of the primary care unit patients presented complications related to diabetes, but less than 30% in the tertiary health care unit and less than 5% in the primary care centers showed the minimum acceptable level of indicators as present. When only patients without adequate diabetes control were evaluated, for whom the most careful treatments and evaluations were expected, no patient in any of the health facilities showed the presence of all quality of care assessments evaluated in this study. According to the ADA recommendations, patients with diabetes mellitus and good glycemic control should have at least two HbA1c tests, and patients who are not well-controlled should undergo the same testing at least four times a year [18]. Around 50% of the diabetic patients at primary care units and nearly one-fifth of the patients at the tertiary health care unit did not show at least two HbA1c measurements over a period of 1 year. Therefore, they were excluded from our final analysis. Another Brazilian study carried out in the southeast region reported similar data, where only 50% of the patients with diabetes mellitus had HbA1c results reported in medical records [13].

It is well known that type 2 diabetes accounts for 80% of nontraumatic lower-limb amputations and shows a high mortality level [20]. To prevent this, regular feet assessments are recommended [21]. In our study, diabetic neuropathy evaluation had very low rates, particularly in primary care. This was similar to another Brazilian study in which less than one-third of patients had undergone feet assessments [22], but this was higher than the data found in an Italian study that evaluated 20,744 patients via the records of 270 physicians. In this study, only 0.9% of the patients were evaluated for neuropathy [23].

For retinopathy, we found very low rates of covered indicators at all the health care units analyzed in this study. The technical limitation of carrying out a fundoscopy/retinography test may be the main reason for these results, as some similar data suggest [24]. Most clinicians refer their patients to an ophthalmologist, but there are not enough of these professionals, as is similar to other low-income countries [25]. Individuals in countries in the highest ophthalmologist availability quartile are less likely to be unaware that they have diabetic retinopathy and less likely to have vision-threatening diabetic retinopathy than individuals who live in countries in the lower ophthalmologist availability quartile [26]. Thus, recognizing that few diabetic patients receive their fundoscopies should let us concentrate efforts on increasing access to ophthalmologists to improve outcomes related to diabetic retinopathy.

Technical limitations due to the lack of a multidisciplinary team may also cause the low rates found for nutritional assessment. Importantly, a full understanding of the situation at each level of health care should be sought; having the personnel available does not imply that these professionals (endocrinologist, nutritionist, nurse, pharmacist, etc.) work together as a team [27, 28].

Nephropathy was the most frequently assessed care quality indicator, with levels similar to those from a study conducted in eight European countries [29]. A reason for this above average performance in relation to the other items is potentially related to how nephropathy was assessed in our study. Creatinine, used to estimate the glomerular filtration rate, was included as a nephropathy assessment criterion, as suggested by the ADA [21]. Because it is a widely available and commonly requested test for patients clinically assessed not only for diabetes, we believe this may limit its validity as a quality indicator for specifically evaluating diabetes patient care.

According to a meta-analysis of macrovascular complications involving 13 cohorts, it is estimated that the relative risk of a cardiovascular event decreases by 18% for each 1% point decrease of HbA1c [30]. Despite the importance of glucose control in reducing this risk, many other cardiovascular risk factors coexist with diabetes and are probably stronger predictors of cardiovascular outcomes than glucose control itself. Smoking, for instance, is an important independent cardiovascular risk factor that may reduce cardiovascular event rates by nearly 50% when discontinued [31, 32]. One of the most relevant disease management items for diabetes patients is the inquiry about tobacco use, considering smoking’s impact as an additional risk factor and the impact of its discontinuation. In our study, we observed very low levels of this inquiry, below 11% in all care levels. Even in multicenter Brazilian studies, much higher levels were previously found (57%) [16]. Another possible explanation for our findings is underreporting because data were retrospectively collected from medical records.

In addition to the suggested regular assessments, adherence to treatment greatly impacts the achievement of proposed therapeutic goals [33, 34]. Patient-centered care and self-management are critical to the effective control of diabetes [6]. At all health care units analyzed in this study, medical records often indicated poor adherence to treatment. In spite of the diverse characteristics in the two primary care units, we found no significant differences in the profile of patients or in the prevalence of care indicators. Despite the higher complexity profile, the higher prevalence of comorbidities and diabetes complications, and the lower levels of patient education at the tertiary care unit, medical records showing poor adherence to treatment were similar to those found in the primary care units.

The higher levels of HbA1c found in the tertiary health care unit patients are probably due to the severity profile and the higher complexity of such patients instead of poor adherence to treatment. In any case, considering these findings could increase the prevalence of care quality indicators for this population to assure the best possible care.

Despite the importance of these results, we must emphasize that this study has some limitations. The retrospective nature of data collection via manual and electronic medical records and the fact that we evaluated health care activity reports (not the execution of the activities) may cause assessment bias in the data. The low quality of reports found in medical records may be one factor accounting for the results. It is also important to point out that this study was conducted at only three local health care centers. As inclusion criterion, patients should have two follow-up HbA1c exams within 1 year for the cohort. This factor may be a limitation as it excludes patients with worse disease control because the two HbA1c measurements were probably associated with better diabetes care; another limitation is the fact that 50% of the primary care patients versus 20% of the tertiary care patients could not participate in the study because they did not have these two annual evaluations. In any case, we believe these results offer important information on how we deliver diabetes care to our patients and on the need for some review and strategy changes to improve health care.

## Conclusions

In conclusion, we see unsatisfactory results in regards to type 2 diabetes mellitus patient care, both at primary and tertiary care units. We hope to contribute to the dialogue among health managers who aim to improve the protocols and care pathways of patients with type 2 diabetes.

## Abbreviations

ADA: American Diabetes Association
ANOVA: analysis of variance
ESF: Estratégia de Saúde da Família
FIPE: Fundo de Incentivo ao Pesquisador
HbA1c: glycated hemoglobina
HCPA: Hospital de Clínicas de Porto Alegre
HDL: high density lipoprotein
LDL-c: low density lipoprotein cholesterol
SPSS: statistical package for the social sciences
STROBE: strengthening the reporting of observational studies in epidemiology
UBS: Unidade Básica de Saúde
